# Insulin signaling in the aging of healthy and proteotoxically stressed mechanosensory neurons

**DOI:** 10.3389/fgene.2014.00212

**Published:** 2014-07-23

**Authors:** Courtney Scerbak, Elena M. Vayndorf, J. Alex Parker, Christian Neri, Monica Driscoll, Barbara E. Taylor

**Affiliations:** ^1^Institute of Arctic Biology, University of Alaska FairbanksFairbanks, AK, USA; ^2^Department of Biology and Wildlife, University of Alaska FairbanksFairbanks, AK, USA; ^3^Department of Neuroscience, CRCHUM, University of MontrealMontreal, QC, Canada; ^4^Laboratory of Neuronal Cell Biology and Pathology, Centre National de la Recherche Scientifique, UMR 8652Paris, France; ^5^Sorbonnes Universités, UPMC Univ Paris 06Paris, France; ^6^Nelson Biological Laboratories, Department of Molecular Biology and Biochemistry, Rutgers, The State University of New JerseyPiscataway, NJ, USA

**Keywords:** neuronal aging, insulin signaling, proteotoxicity, Huntington's Disease, *Caenorhabditis elegans*

## Abstract

Insulin signaling is central to cellular metabolism and organismal aging. However, the role of insulin signaling in natural and proteotoxically stressed aging neurons has yet to be fully described. We studied aging of *Caenorbaditis elegans* mechanosensory neurons expressing a neurotoxic expanded polyglutamine transgene (polyQ128), or lacking this proteotoxicity stressor (polyQ0), under conditions in which the insulin signaling pathway was disrupted by RNA interference (RNAi). We describe specific changes in lifespan, mechanosensory neuronal morphologies, and mechansensory function following RNAi treatment targeting the insulin signaling pathway. Overall, we confirmed that transcription factor DAF-16 is neuroprotective in the proteotoxically stressed model, though not strikingly in the naturally aging model. Decreased insulin signaling through *daf-2* RNAi improved mechanosensory function in both models and decreased protein aggregation load in polyQ128, yet showed opposing effects on accumulation of neuronal aberrations in both strains. Decreased *daf-2* signaling slightly enhanced mechanosensation while greatly enhancing branching of the mechanosensory neuron axons and dendrites in polyQ0 animals, suggesting that branching is an adaptive response in natural aging. These effects in polyQ0 did not appear to involve DAF-16, suggesting the existence of a non-canonical DAF-2 pathway for the modulation of morphological adaptation. However, in polyQ128 animals, decreased *daf-2* signaling significantly enhanced mechanosensation while decreasing neuronal aberrations. Unlike other interventions that reduce the strength of insulin signaling, *daf-2* RNAi dramatically redistributed large polyQ128 aggregates to the cell body, away from neuronal processes. Our results suggest that insulin signaling strength can differentially affect specific neurons aging naturally or under proteotoxic stress.

## Introduction

The insulin/insulin-like growth factor (IGF) signaling pathway is involved in longevity and stress response across species (Broughton and Partridge, 2009). Signaling through this evolutionarily conserved pathway can promote longevity through increased expression of cellular stress and metabolism genes, including those encoding stress-response, chaperone, and antioxidant proteins (Kenyon, 1993; Hsu et al., 2003; Taguchi et al., 2007; Cohen et al., 2009). The progression of neurodegenerative disorders, such as Huntington's, Alzheimer's, and Parkinson's diseases, has been linked to insulin signaling in both invertebrate and mammalian model systems (Dillin and Cohen, 2011). In addition, decreased insulin signaling has protective effects against neurodegenerative-associated proteotoxicity across species (Freude et al., 2009; Killick et al., 2009).

**Figure 1:**
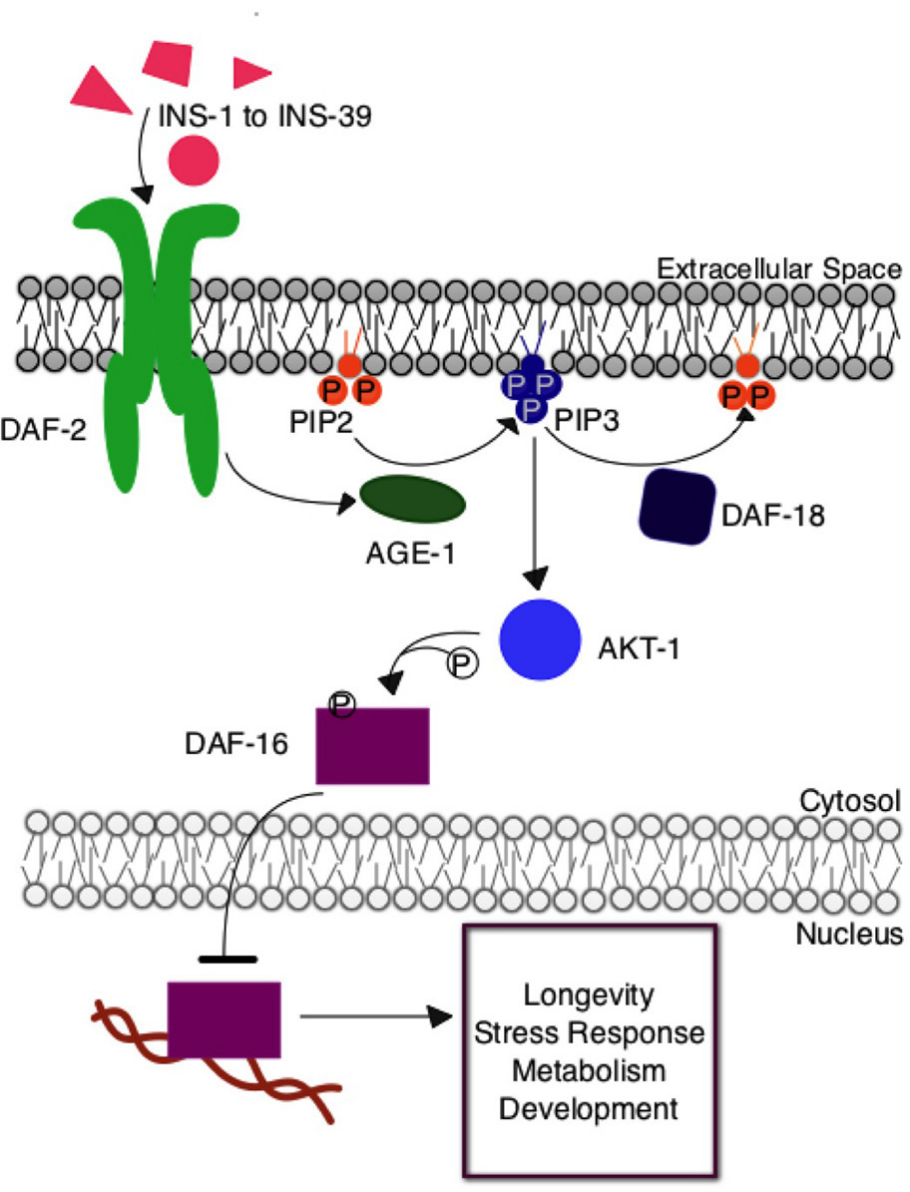
**The insulin signaling pathway in *Caenorhabditis elegans***. Upon insulin ligand binding to the DAF-2 insulin receptor in *C. elegans*, AGE-1 (homolog to mammalian PI3-K) is activated and converts PIP_2_ to PIP_3_ at the cellular membrane. PIP_3_ activates the central kinase AKT-1 (ortholog to mammalian PKB), which phosphorylates DAF-16, the *C. elegans* FOXO transcription factor, preventing its entry into the nucleus where it would otherwise regulate the expression of genes contributing to longevity, stress response, and metabolism. The DAF-18 (homolog to mammalian PTEN) phosphatase negatively regulates the system by decreasing the amount of PIP_3_ present at the membrane by reconverting it to PIP_2_.

The *Caenorhabditis elegans* insulin signaling pathway is regulated by insulin-like signaling ligands, INS-1 through INS-39, that modulate the activity of the DAF-2 tyrosine kinase receptor (Figure 1). DAF-2 is orthologous to the mammalian insulin/IGF receptor. This receptor activates a protein kinase signaling pathway, which, through phosphorylation of downstream transcription factor DAF-16 by AKT protein kinase, regulates functions similar to receptor kinases in the insulin signaling pathway in humans. Under high signaling conditions, DAF-16 is phosphorylated to prevent nuclear entry and hence transcription. Under low signaling conditions, DAF-16 is free from inhibitory phosphorylation and can regulate the expression of many different genes contributing to metabolism and physiological defense and homeostasis responses (Mukhopadhyay et al., 2006). This insulin signaling pathway has many branch points, including AKT, and there is some variation in the identities of proteins involved in nematodes, flies, and mice, three organisms in which the role of insulin signaling in aging has been investigated. Nevertheless, this central, conserved insulin signaling pathway is critical for appropriate cellular metabolism and maintenance of overall organismal health.

Various mutations in genes modulating the *C. elegans* insulin signaling pathway have been shown to directly regulate lifespan. For example, *daf-2* insulin receptor mutants live twice as long as wildtype animals (Kenyon, 1993), *age-1* PI3K mutants live longer than wildtype animals (Friedman and Johnson, 1988), and the lack of the *daf-16* FOXO transcription factor gene shortens lifespan (Oh et al., 2006). Longevity effects can result from limiting insulin signaling in only the neurons or intestinal cells (Libina et al., 2003). In mammals, the effect of reduced insulin signaling on overall health and lifespan is complex, but combined evidence from many studies points to the potential of insulin signaling reduction to extend lifespan (Taguchi and White, 2008). In mice, reduced neuronal or whole animal expression of IRS2, a kinase activated by the insulin receptor, increases lifespan (Taguchi et al., 2007). In humans, various studies have detected an association between longevity and single nucleotide polymorphisms in genes involved in insulin signaling, including the DAF-16-related transcription factor inhibited by insulin signaling (FOXO3A), the insulin receptor (IGF1R), and a central protein kinase (AKT-1) (Newman and Murabito, 2013). Excellent reviews comparing insulin signaling in various model systems have been published (Taguchi and White, 2008; Broughton and Partridge, 2009; Neri, 2012).

*C. elegans* exhibit many important neuronal components found in humans, including, but not limited to, neurotransmitters, ligand receptors, and ion channels; thus, these animals are a powerful model for studying neuronal aging and neurodegeneration *in vivo*. Neurodegenerative disorders, such as Huntington's, Alzheimer's, and Parkinson's diseases, result in the progressive loss of structure and function of neurons with age. Of these protein aggregation-associated neurodegenerative diseases, Huntington's disease is caused by the expansion of CAG trinucleotide repeats in the huntingtin gene, which results in an expansion of the length of polyglutamine residues at the N-terminus of the huntingtin protein. Expanded polyglutamine repeats in mutated huntingtin lead to neuronal protein aggregation, impairments in movement and cognitive function, and psychological disorders. Multiple Huntington's disease model strains of *C. elegans* have been developed (Faber et al., 1999; Parker et al., 2001, 2005; Morley et al., 2002). In the model strain used in this study, touch-receptor neuron-specific expression of a transgene encoding the first 57 amino acids of human huntingtin with 128 polyglutamine repeats impairs function, without neuronal death (Parker et al., 2001, 2005). Thus, this model may feature conserved events associated with dysfunction that typify early disease stages in humans.

Aging is the primary risk factor for multiple neurodegenerative diseases, yet the intersection of natural neuronal aging and neurodegenerative states is not well understood. As a consequence of sensing and responding to the environment, the nervous system is known to play a role in physiological aging (Alcedo et al., 2013). In normal, healthy aging, *C. elegans* mechanosensory and other neuron classes develop morphological aberrations, including new outgrowths from the soma, novel process branching, and dendritic restructuring (Pan et al., 2011; Tank et al., 2011; Toth et al., 2012). Neuronal insulin signaling appears to be involved in this natural aging process; the link between normal aging and decline under disease conditions is relatively unexplored. To address this relationship, we studied and compared morphological features of aging mechanosensory neurons with and without a neurotoxic expanded polyglutamine transgene, under conditions in which genes of the canonical insulin signaling pathway were disrupted by neuron-targeted RNA interference (RNAi). Our findings suggest that insulin signaling strength can differentially affect specific neurons aging naturally or under conditions of disrupted proteostasis. Under conditions of polyglutamine expansion stress, insulin receptor DAF-2 appears to act through DAF-16/FOXO. However, under conditions of normal aging, DAF-2 activates a non-canonical pathway that acts independently to induce neuroprotection.

## Materials and methods

### Strains

The *C. elegans* Huntington's disease model strain used was derived from two previously engineered strains. Strain ID1 (*igls1* [*P_mec−7_yfp, P_mec-3_htt57Q128::cfp, lin-15(+)*]) (polyQ128) contains the first 57 amino acids of human huntingtin fused to CFP-labeled expanded polyglutamine tract (Q128) expressed in the 6 mechanosensory neurons as well as in PVD and FLP neurons (Parker et al., 2001). These polyQ128 animals show functional deficiencies in touch response and accumulation of huntingtin protein aggregates in mechanosensory neurons, without cell death (Parker et al., 2001). Strain TU3270 (*uIs57* [*P_unc-119_SID-1, P_unc-119_yfp, P_mec-6_mec-6*]) overexpresses the transmembrane channel SID-1 pan-neuronally, allowing the dsRNA from RNAi treatment to enter all neurons (Calixto et al., 2010). TU3270 and ID1 were crossed to generate ZB4062 *igIs1*[*P_mec-7_yfp, P_mec-3_htt57Q128::cfp, lin-15(+)*]; *uIs57* [*P_unc-119_SID-1, P_unc-119_yfp, P_mec-6_mec-6*], a polyQ128-expressing strain with neurons susceptible to RNAi. We also crossed a healthy transgenic model, ZB154 (*zdIs5* [*P_mec-4_GFP, lin-15(+)*]), to TU3270 to generate ZB4064 *zdIs5* [*P_mec-4_GFP, lin-15(+)*]; *uIs57* [*P_unc-119_SID-1, P_unc-119_yfp, P_mec-6_mec-6*] to render mechanosensory neurons susceptible to RNAi treatment (polyQ0).

### Worm maintenance

Standard methods were used for strain maintenance, bacterial culturing, and animal manipulation (Brenner, 1974). Stock animals were cultured at room temperature (about 22°C) on nematode growth media (NGM) agar plates seeded with live bacteria (*E. coli* strain OP50-1).

### RNA interference treatments

We prepared RNAi plates using 4× concentrated live HT115 *E. coli* bacteria from the Ahringer Library induced at room temperature for 2 days on agar plates. For each batch of RNAi experiments, we performed a control experiment comparing the amount of nerve ring fluorescence knockdown following GFP treatment to empty vector (L4440) in age-matched animals to confirm neuronal RNAi sensitivity of the strain used (Supplemental Figure 1). Only experiments that showed a significant (unpaired *t*-test, *p* < 0.05) knockdown of GFP were used for further RNAi studies. We performed RNAi treatments at 25°C protected from light with age-synchronous populations created using timed egg lay. To perform each egg lay, adult worms laid eggs on each of the described RNAi treatment plates for 4 h. Animals in all RNAi experiments were transferred by hand each day of adulthood to fresh RNAi plates. We performed and analyzed RNAi experiments “blinded” to the intervention so that the experimenter was not aware of the genetic identity of the RNAi treatment given to each population of animals. We repeated each experiment at least 3 times. We selected day 5 of adulthood as a time point for analysis in the following experiments based on Toth et al. (2012) who reported a significant difference in mechanosensory neuronal morphology between day 1 and day 5 of adulthood.

### Lifespan analysis

Following the production of age-synchronous populations, we transferred approximately 50 animals from each RNAi treatment group everyday of adulthood to fresh, seeded RNAi treatment small plates and checked for survival by visual observation or gentle prodding with a platinum wire. Animals with protruding intestines, those that bore live young, or that crawled off the plates were censored. Survival experiments always included all 6 RNAi treatment groups (L4440 empty vector, *daf-2, age-1, daf-18, akt-1, daf-16*) at once for, usually, one strain at a time, and were repeated at least twice. We used Kaplan–Meier log-rank survival statistics to analyze differences in mean survival between RNAi treatment groups and *p* < 0.05 was noted as significant.

### Mechanosensory response assay

**Figure 2:**
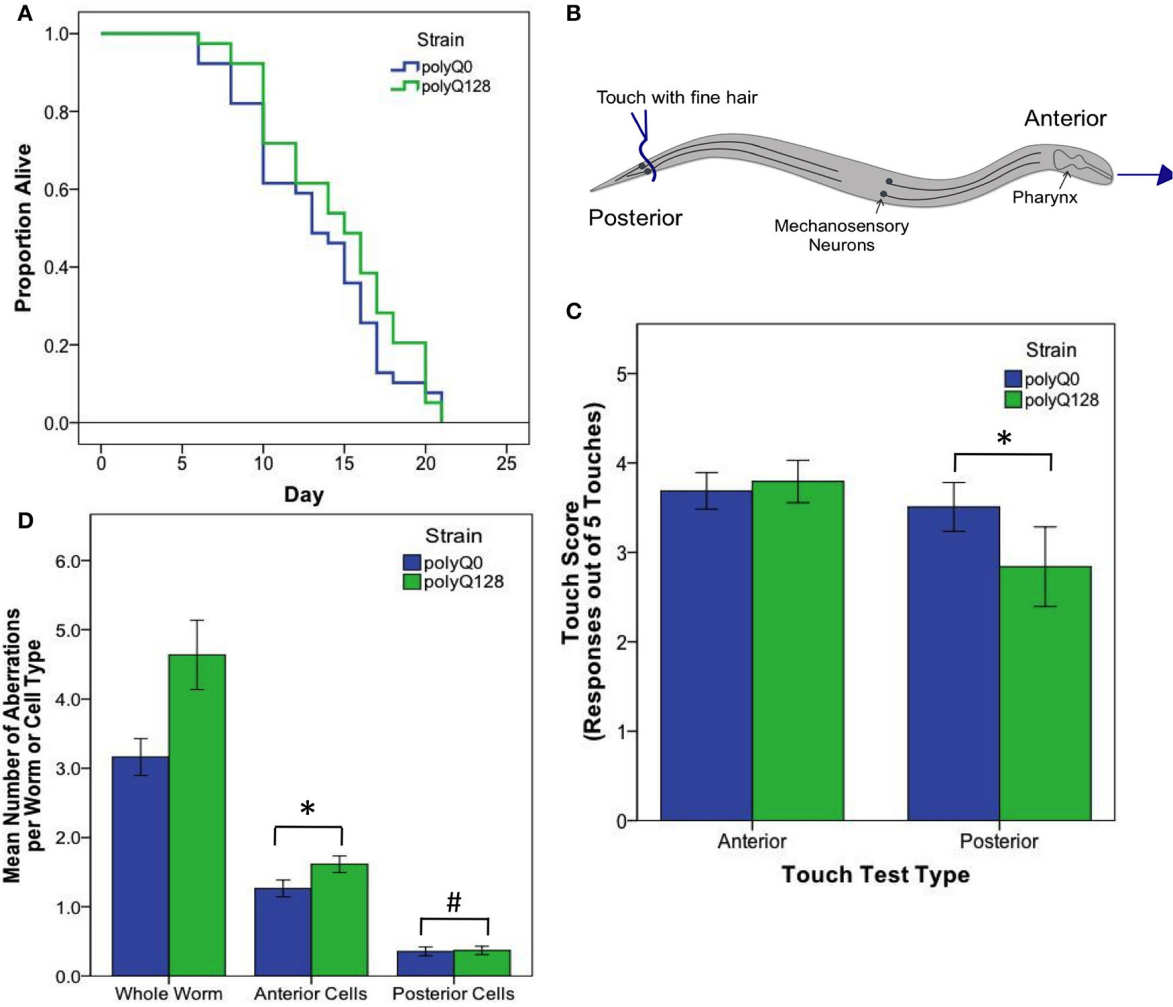
**PolyQ0 and polyQ128 exhibit basal differences in mechanosensory neuron morphology and function without altered lifespan. (A)** Kaplan–Meier survival curves for one representative survival experiment shown for empty vector (L4440) treated polyQ0 and polyQ128 animals. Mean and median lifespan do not differ between the two strains. **(B)** Schematic of posterior touch test; an animal is touched at the tail and normally moves forward, gaining 1 positive response to touch (out of 5). For anterior touch test, animals are touched at the head and scored for backwards movement. Mechanosensory neurons (PLMs and ALMs) and pharynx shown. **(C)** Anterior and posterior touch test scores for empty vector control individuals. Each bar represents the mean number of positive responses out of 5 to soft touch. **(D)** Total number of aberrations (e.g., number of outgrowths and branches and presence of abnormal cell body and punctae) per worm and cell type for each empty vector control group. Whole worm bars represent the mean values of the sum of all 4 neurons scored (ALML, ALMR, PLML, and PLMR) for each individual. Anterior and posterior bars represent the mean values for individual anterior (ALML or ALMR) or posterior (PLML or PLMR) neurons. ^*^Denotes significance of *p* < 0.01 and ^#^denotes significance of *p* < 0.10 following Mann–Whitney U comparison. Each bar shows mean ± SE for *N* = 69 (polyQ0) and *N* = 84 (polyQ128) animals.

We generated synchronous populations as described above, maintained cultures at 25°C, and transferred each day of adulthood to fresh RNAi treatment plates. On day 5 of adulthood we scored individuals for motility class. Individuals were grouped into 3 classes: A class indicates normal, voluntary sinusoidal movement, B class indicates locomotion following gentle prodding, and C class indicates inability to locomote. We then tested individuals for their ability to respond to touch by gently touching alternatively on the anterior and posterior end with an eyelash pick, 5 times each (Figure 2B). Animals responded either by moving (or attempting to move) in the opposite direction of the touch or by showing no movement. We scored animals (0–5) based on the number of positive responses to touch out of 5 touches at the anterior and 5 touches at the posterior. We also recorded the mobility of each animal. We then imaged the mechanosensory neurons of the tested individuals as described below.

### Neuronal morphology imaging

Following the mechanosensory response assay on day 5 of adulthood, we mounted animals between 2 cover slips using 2 μL of 36% w/v Pluronic™ solution dissolved in water and imaged using the 20× objective of an Axiovert S100 inverted fluorescent microscope. Using constant microscope settings, we collected images of the 6 mechanosensory neurons of each individual and their associated huntingtin protein aggregates, if present, using FITC and CFP filters, respectively. We discarded animals after imaging, so data presented is cross-sectional rather than longitudinal. We repeated mechanosensory response assays and neuronal morphology imaging for each strain and RNAi treatment group at least 3 times. We examined the images of 6 mechanosensory neurons of each individual for morphological aberrations, namely cell body outgrowths, cell body guidance errors, and process branching (Toth et al., 2012).

### Protein aggregate quantification and data analysis

For polyQ128 experiments, we analyzed huntingtin aggregates (fused with CFP) with ImageJ, using a custom macroinstruction that includes quantification of the total area of aggregates seen in each cell. For comparisons of mechanosensory response and neuronal morphology between empty vector control polyQ0 and polyQ128 strains, we used a Mann–Whitney U comparison. For the mechanosensory response assays, neuronal morphology imaging, and protein aggregate counts following RNAi treatments, we used a generalized linear model with a log link function (Poisson regression) and Wald tests for significance of treatment effects. For total aggregate area measurements, we used a One-Way ANOVA with Tukey's pairwise comparisons. SPSS (Version 20) statistical software was used to perform, the analyses. A *p*-value of less than 0.05 was considered statistically significant. Values presented in the text represent mean ± standard error.

## Results

### Neuron and systemic RNAi knockdown of insulin signaling proteins alters polyQ0 and polyQ128 *C. elegans* lifespan

To initiate analysis of the influence of key insulin signaling pathway genes on normal aging and polyQ128-induced neuronal deficits in mid-adult life, we constructed *C. elegans* strains by genetic crosses with fluorescent mechanosensory (or touch) neuron reporters that express the *sid-1* double stranded (ds) RNA transporter pan-neuronally. This *sid-1* compensates for the lack of a neuronal dsRNA transporter, enabling genes expressed in neurons to be targeted by RNAi (Calixto et al., 2010). Moreover, *sid-1* overexpression in neurons can diminish non-neuronal RNAi effects, such that the *sid-1(+)* neurons act as a sink for double stranded RNA (Calixto et al., 2010). We studied one strain that was free of proteotoxic stress (hereafter referred to as polyQ0), and one that expresses the first 57 amino acids of human huntingtin protein with expanded polyglutamines fluorescently labeled with CFP (polyQ128) (Parker et al., 2001). In these strains, we can measure neuronal function via mechanosensory touch response assays, visualize neuronal morphology structures, and directly observe polyQ128 aggregates. We first confirmed that polyQ0 and Huntington's disease model (polyQ128) strain neurons were sensitive to RNAi treatment by feeding as detected by GFP knockdown in the nerve ring (Supplemental Figure 1). Indeed, GFP knockdown was efficient in both strains, supporting that our intended studies on insulin signaling pathway genes could effectively target mechanosensory neurons.

Interestingly, healthy polyQ0 and proteotoxically stressed polyQ128 empty vector (L4440) treated animals exhibited similar, not significantly different, mean lifespan (Figure 2A). Thus, increased polyglutamine load in mechanosensory neurons does not confer decreased lifespan, consistent with previous work showing mechanosensory neurons are dispensable for viability and lifespan (Chalfie et al., 1985). However, empty vector treated control polyQ128 animals do exhibit signs of abnormal function as measured by motility, touch response, and accumulation of mechanosensory neuronal aberrations (Table 1, Figures 2C,D), agreeing with previous work (Parker et al., 2001). Specifically, polyQ128 posterior touch response is significantly decreased (Mann–Whitney U, *p* < 0.01) and neuronal aberrations are increased at the whole worm (Mann–Whitney U, *p* < 0.01), anterior cells (Mann–Whitney U, *p* < 0.01), and posterior cells level (Mann–Whitney U, *p* = 0.04) when compared to age-matched polyQ0 individuals. This suggests that increased polyglutamine load in the mechanosensory neurons negatively affects the function and healthspan of polyQ128 animals.

**Table 1:** **Effect of RNAi treatment on polyQ0 and polyQ128 motility**.

**Strain**	**RNAi treatment**	**Number per motility class**			**Total *N***
		***A***	***B***	***C***	
polyQ0	Empty vector	65	4	0	69
	*daf-2*	53	0	0	53
	*age-1*	67	4	0	71
	*daf-18*	47	2	0	49
	*akt-1*	50	1	0	51
	*daf-16*	48	3	0	51
polyQ128	Empty vector	69	15	0	84
	*daf-2*	87	6	0	93
	*age-1*	77	18	0	95
	*daf-18*	47	0	0	47
	*akt-1*	31	15	1	47
	*daf-16*	51	20	1	72

*Number of individuals falling into A, B, or C class motility for each treatment group is shown. A class indicates normal, voluntary sinusoidal movement, B class indicates locomotion following gentle prodding, and C class indicates inability to locomote. Overall, polyQ128 animals have higher incidence of B class motility than polyQ0. Only polyQ128 daf-18(RNAi) results in a significant difference in motility compared to control*.

**Figure 3:**
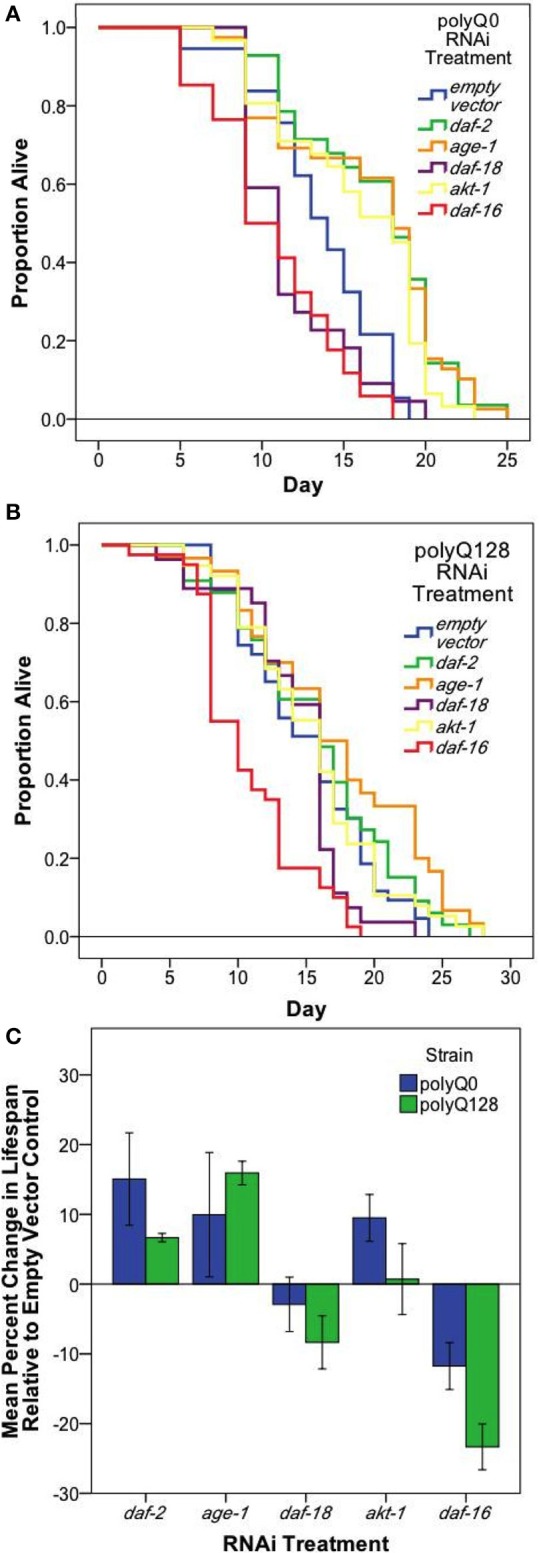
**Neuronal and systemic insulin signaling mediates lifespan of both healthy (polyQ0) and proteotoxically stressed (polyQ128) animals**. Kaplan–Meier survival curves for one representative survival experiment shown for polyQ0 **(A)** and polyQ128 **(B)** animals with RNAi knockdown of the indicated insulin signaling pathway gene. Mean lifespan, sample size, and significance reported as Replicate 1 for each strain in Table 2. **(C)** Mean percent change in lifespan with RNAi treatment for polyQ0 and polyQ128 animals pooled across biological replicates. Only *daf-16* RNAi showed a significant impact on lifespan for polyQ128 animals when compared to polyQ128 empty vector controls (23.3 ± 4.1% for 3 replicates of 45–50 animals). Each bar represents mean ± SE.

Because several previously published mutant and RNAi experiments did not utilize *sid-1* enhanced neuronal RNAi targeting, and thus would not have assayed neuronal knockdown effects, we also confirmed RNAi effects on longevity under the conditions we used for our studies. We found that knockdown of insulin signaling pathway genes in neurons and other tissues altered healthy polyQ0 life as previously reported, with *daf-2*, *age-1*, and *akt-1* RNAi interventions lengthening lifespan, and *daf-16* RNAi shortening it (Figure 3A and Table 2). Insulin signaling pathway interventions in the polyQ128 strain (*daf-2*, *age-1*) similarly increased mean lifespan, while *daf-16* RNAi treatment decreased mean lifespan (Figure 3B and Table 2). In the 3 biological replicates of *akt-1* RNAi in polyQ128 animals, lifespan impact was variable.

**Table 2:** **Effects of RNAi treatment targeting insulin signaling on polyQ0 and polyQ128 animals' mean lifespan**.

**Strain**	**RNAi treatment**	**Replicate 1**		**Replicate 2**		**Replicate 3**	
		***N***	**Mean lifespan days ± *SE***	***N***	**Mean lifespan days ± *SE***	***N***	**Mean lifespan days ± *SE***
polyQ0	Empty vector	50	13.7 ± 0.56	60	14.3 ± 0.58	55	13.5 ± 0.66
	*daf-2*	40	16.8 ± 0.83^*^	*N/A*	*N/A*	48	16.3 ± 1.04^*^
	*age-1*	51	16.3 ± 0.82^*^	59	16.9 ± 0.61^*^	*N/A*	*N/A*
	*daf-18*	30	11.7 ± 0.70	60	14.8 ± 0.57	50	13.5 ± 0.62
	*akt-1*	40	15.8 ± 0.79^*^	60	16.2 ± 0.52^*^	48	13.4 ± 0.50
	*daf-16*	46	11.0 ± 0.61^*^	60	12.1 ± 0.53^*^	51	12.9 ± 0.68
polyQ128	Empty vector	50	15.2 ± 0.71	50	15.1 ± 0.61	56	13.4 ± 0.55
	*daf-2*	51	16.1 ± 0.94	50	16.2 ± 0.89^*^	*N/A*	*N/A*
	*age-1*	40	17.9 ± 1.01^*^	*N/A*	*N/A*	59	15.0 ± 0.89^*^
	*daf-18*	38	15.6 ± 0.79	50	14.5 ± 0.66	59	11.4 ± 0.42^*^
	*akt-1*	47	15.8 ± 0.79	25	14.4 ± 1.15	56	11.4 ± 0.44^*^
	*daf-16*	48	10.9 ± 0.60^*^	50	12.8 ± 0.63^*^	61	10.4 ± 0.39^*^

Mean lifespan in days ± SE of each RNAi treatment replicate for the polyQ0 and polyQ128 strains are shown. Each replicate of 6 treatments (empty vector control, DAF-2, AGE-1, DAF-18, AKT-1, and DAF-16) was run one strain at a time. Entries with

* *are significantly different (p < 0.05) from the strain- and replicate-matched empty vector control following Kaplan–Meier log-rank survival statistics. Replicate with missing data (N/A) was censored from experiment*.

To further address whether there might be differences in general viability between polyQ0 and polyQ128 strains, we compared percent change in mean lifespan between polyQ0 and polyQ128 animals for each RNAi treatment group. We found no significant differences in relative percent changes between healthy transgenic and polyQ128 animals for the same RNAi treatment (*t*-tests, *p* > 0.05), except for *daf-16* RNAi, which approached significance in the polyQ128 background (Figure 3C; *p* = 0.07). We also measured endogenous reactive oxygen species (ROS) as detected by the membrane permeable 2′, 7′-Dichlorofluorescin diacetate (DCF-DA, Sigma) following RNAi treatments and found that RNAi treatment targeted at insulin signaling pathway proteins from embryo did not consistently significantly affect young adult (day 1) endogenous ROS levels in both healthy transgenic and polyQ128 strains (data not shown). However, *daf-16* RNAi treatment through mid-life in polyQ128 worms significantly increased endogenous ROS levels across all biological replicates.

We conclude that RNAi interventions in our polyQ0 and polyQ128 strains are efficacious and exert similar general influences on aging biology in polyQ0 and polyQ128 strains. Disruption of a small set of sensory neurons by the proteotoxic stress of polyQ128 is not sufficient to grossly impair whole animal function, although there are some functional impairment and increased morphological aberrations. However, we note that in middle-aged adults, *daf-16* RNAi is associated with elevated ROS levels and decreased mid-life viability specifically in the polyQ128 strain, raising the possibility that the combination of small scale neuronal proteostasis disruption can influence entire organism decline when DAF-16-dependent defenses are impaired.

### Decreased insulin signaling through RNAi knockdown influences healthy mechanosensory neuron morphology and function

**Figure 4:**
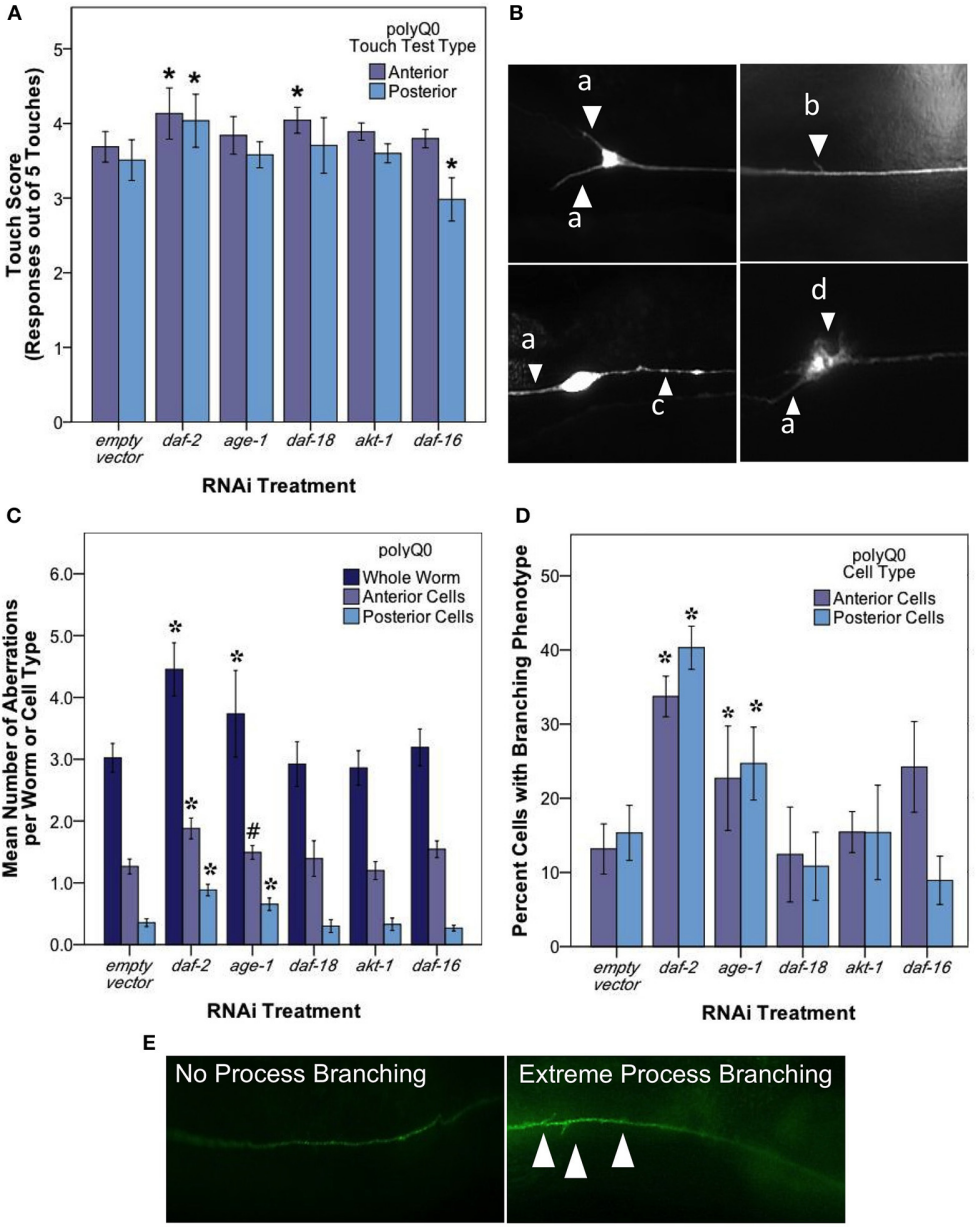
***daf-2* RNAi mediates mechanosensory neuron morphology and function in polyQ0 animals. (A)** Anterior and posterior touch test scores of polyQ0 animals following RNAi treatment. Each bar represents the mean number of positive responses to 5 soft touches. **(B)** Representative examples of outgrowths (a), branching (b), punctae (c), and abnormal cell body (d) phenotypes seen in aging mechanosensory neurons of polyQ0 and polyQ128 individuals. **(C)** Mean number of neuronal aberrations per whole animal (sum of all 4 neurons scored in an individual) and individual cell type (anterior [ALML or ALMR] and posterior [PLML or PLMR]). **(D)** Percentage of neurons with branching phenotype for each RNAi treatment. Neurons with this phenotype may have more than one branch (data not shown). **(E)** Representative images of mechanosensory neuron processes and process branching (white arrows) in polyQ0 animals. ^*^Denotes significance of *p* < 0.01 and ^#^denotes significance of *p* < 0.10 relative to appropriate empty vector control following a generalized linear model with a log link function (Poisson regression) and Wald tests for significance of treatment effects. Each bar represents mean ± SE for *N* = 49–70 animals.

Previous studies on morphological aging of mechanosensory neurons indicated that manipulation of the insulin signaling pathway can change accumulation of neuronal aberrations (Pan et al., 2011; Tank et al., 2011; Toth et al., 2012). These studies differed in details of methods and outcomes, and did not address *akt-1* or *daf-18* activities. Moreover, these studies left open the question of how morphological features relate to function. To address these gaps and discrepancies, we measured neuronal morphology and touch response as a surrogate for mechanosensory function when components of the insulin signaling pathway were knocked down in polyQ0 animals. We performed touch response tests (Figure 4A) and then imaged mechanosensory neurons of characterized animals for changes in morphology (Figures 4B,C) at mid-life (day 5 of adulthood; described in detail in Materials and Methods). Total neuronal aberrations include all morphological aberrations observed in an individual or cell type, including soma outgrowths, abnormal cell somas, process branching, and process punctae (Figure 4B).

Our analysis of touch sensory function revealed that both anterior and posterior touch responses in *daf-2* RNAi were better than empty vector at day 5 of adult life (Figure 4A; Wald test, *p* < 0.01 for anterior and posterior scores). *daf-16* RNAi preferentially decreased posterior touch (*p* < 0.01), revealing an interesting difference between anterior and posterior mechanosensory neurons. We found that RNAi knockdown of *age-1*, *daf-18*, and *akt-1* did not alter touch responses as compared to empty vector.

A striking result from our analysis of morphological aberrations at day 5 of adulthood is that *daf-2* RNAi increased the occurrence of total neuronal aberrations as compared to control (from 3.2 ± 0.23 to 4.7 ± 0.26 per individual) in both anterior and posterior neurons (Figure 4C). We found that this increase was driven exclusively by process branching, which was the only specific neuronal aberration observed to change with *daf-2* RNAi (Figure 4D; from 12.8 ± 3.40 to 32.7 ± 3.80% in anterior cells and 14.5 ± 3.20 to 39.4 ± 3.70% in posterior cells). *age-1* RNAi also increased total aberrations (from 3.2 ± 0.23 to 4.4 ± 0.23 per individual) (Figure 4C) and novel branching in anterior neurons (Figure 4D; from 12.8 ± 3.40 to 25.3 ± 3.40% in anterior cells and 14.5 ± 3.20 to 26.7 ± 3.30% in posterior cells), whereas *daf-18*, *akt-1*, and *daf-16* interventions did not induce statistically significant changes in overall aberrations or in the hyper-branching phenotype. Our findings suggest that *daf-2* disruption, and lowered insulin signaling could differentially effect different morphologies, and raise the question as to whether branching might be an indication of a neuroprotective response (see Discussion).

### Touch response, mechanosensory neuronal morphology, and protein aggregate accumulation are affected by insulin signaling in a model strain of huntington's disease pathogenesis

We next examined functionality and morphological aberrations in the polyQ128 strain in which mechanosensory neurons are exposed to a chronic proteotoxic stress that promotes early dysfunction (Parker et al., 2001). As previously noted, empty vector treated polyQ128 animals exhibit increased aberrant neuronal morphology and decreased touch response compared to age-matched, empty vector treated wildtype animals (Figures 3C,D).

**Figure 5:**
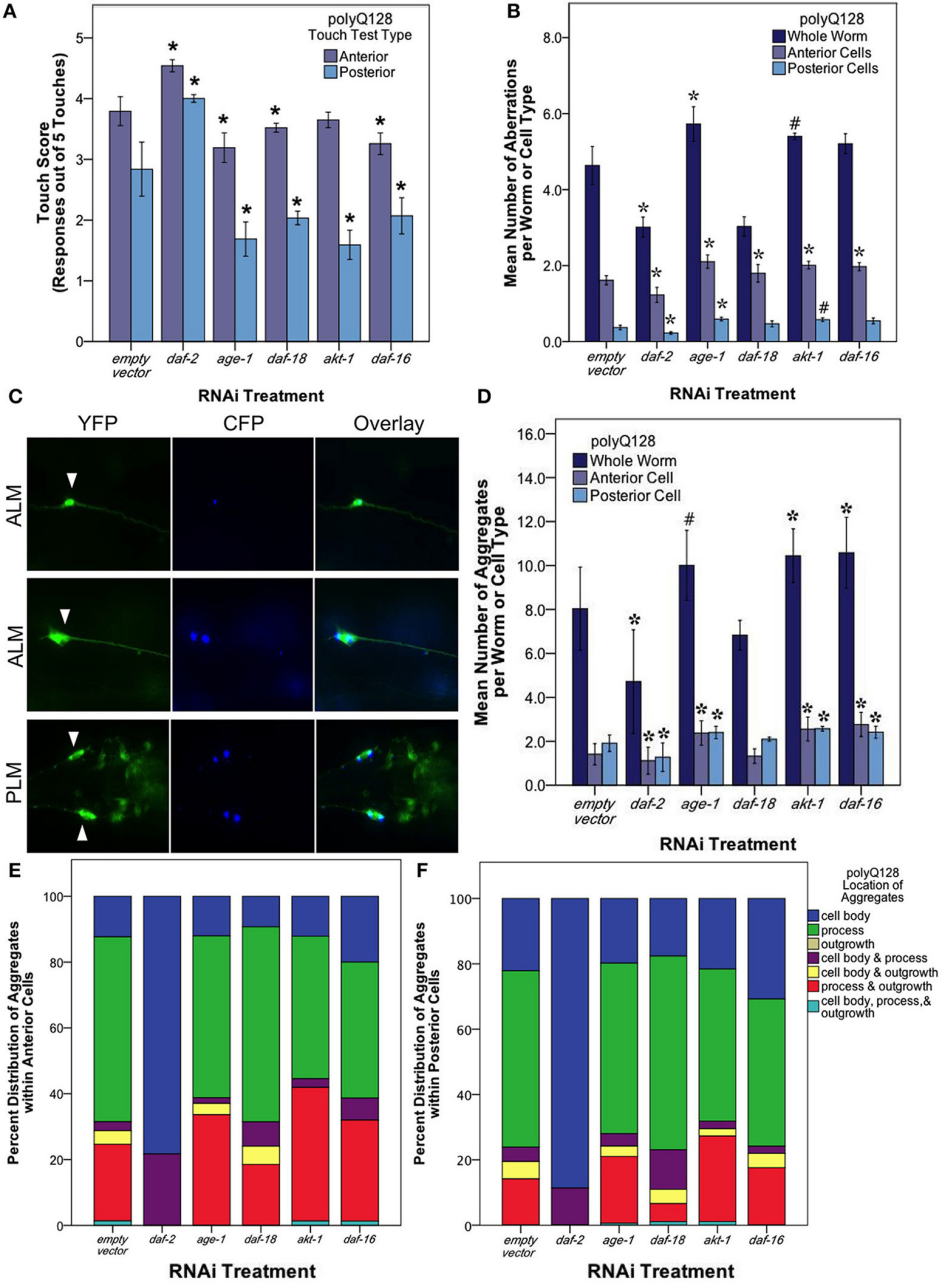
**The insulin signaling pathway mediates mechanosensory neuron morphology and function in polyQ128 animals. (A)** Anterior and posterior touch test scores, representing the number of positive responses to 5 soft touches, following RNAi treatment for indicated insulin signaling pathway gene of polyQ128 animals. **(B)** Mean number of neuronal aberrations per whole animal (sum of all 4 neurons scored in an individual) and individual cell type (anterior [ALML or ALMR] and posterior [PLML or PLMR]). **(C)** Representative image of polyQ128 aggregate (CFP, blue panels) accumulation within anterior (ALM) and posterior neurons (PLM) (YFP, green panels). White arrows in YFP panels indicate the location of the cell body of each neuron. **(D)** The effect of RNAi treatment on the number of distinct CFP-labeled extended polyglutatmine huntingtin protein aggregates in whole animal and anterior and posterior neurons of polyQ128 animals. **(E)** Sub-cellular localization of poyQ128 aggregates within anterior neurons of polyQ128 animals that contained aggregates. **(F)** Location of polyQ128 aggregates in posterior neurons of polyQ128 animals. ^*^Denotes significance of *p* < 0.01 and ^#^denotes significance of *p* < 0.10 relative to appropriate empty vector control following generalized linear model analysis with a log link function (Poisson regression) and Wald tests for significance of treatment effects. Each bar represents mean ± SE.

We tested touch response in polyQ128 animals subjected to RNAi for insulin signaling pathway components (Figure 5A). *daf-2* RNAi had a neuroprotective effect on both anterior (Wald test, *p* < 0.001) and posterior touch sensitivity (Wald test, *p* < 0.001). Interestingly, however, all other RNAi knockdown interventions (*age-1*, *daf-18*, *akt-1*, and *daf-16*) had generally deleterious effects on touch sensitivity in the polyQ128 background, with a particularly significant change in posterior touch response in polyQ128 animals compared with empty vector (L4440).

We then examined neuronal morphology in the polyQ128 strain following RNAi of the insulin signaling pathway genes (Figure 5B). We found that numbers of aberrations in the polyQ128 strain were reduced upon *daf-2* RNAi when compared to empty vector (from 4.78 ± 0.21 to 2.94 ± 0.15 per individual, Wald test, *p* < 0.001). In contrast, *age-1* RNAi, modestly increased aberrations (from 4.3 ± 0.2 to 5.4 ± 0.1 per individual), as did *akt-1* and *daf-16* interventions in anterior mechanosensory neurons. Thus for polyQ128-expressing neurons, morphological aberrations generally inversely correlate with function: low abnormality abundance corresponds to enhanced mechanosensory function.

In polyQ128 animals, the huntingtin:polyQ128 protein is fused with CFP and can be visualized as aggregates in our strain (Figure 5C). We therefore also examined the number and size of fluorescent aggregates in mechanosensory neurons following RNAi knockdown of insulin signaling components (Figure 5D). Under conditions of *daf-2* RNAi we found that mean numbers of aggregates were lowered compared to wildtype (Wald test, *p* < 0.01 for whole individuals, anterior cells, and posterior cells). Mean aggregate area was unchanged for *daf-2* RNAi (Supplemental Figure 2), suggesting that less aggregated protein persists in mid-life mechanosensory neurons when DAF-2 signals are reduced. Conversely, we found modest increases in aggregate number and size for *age-1*, *akt-1*, and *daf-16* knockdown (Figure 5D and Supplemental Figure 2). Importantly, Parker et al. (2005) showed no difference in huntingtin expression of the polyQ128 animals in *age-1* and *daf-16* genetic mutants. This suggests that our findings of altered protein aggregation in the polyQ128 strain is likely not due to changed huntingtin expression, but rather is more likely attributed to cellular responses of the expanded polyglutamine protein (Figure 5).

Interestingly, we noted a striking difference in the localization of polyQ128 protein aggregates in posterior and anterior cells for *daf-2* RNAi (Figures 5E,F). In *daf-2* RNAi treated animals, 80–90% of the detected aggregates localized in the cell body of anterior and posterior cells whereas other insulin signaling pathway knockdowns were associated with a majority (50–60%) of aggregates localized within the process of these cells, similar to empty vector controls. We also note that aggregates were never observed in outgrowths without also being present in the cell body. This dramatic difference of aggregate localization in *daf-2* RNAi animals suggests that subcellular distribution of protein aggregates is regulated by a DAF-2 non-canonical pathway.

### RNAi knockdown of *daf-2* returns polyQ128 mechanosensory neuron morphology to healthy levels

At the whole worm and anterior and posterior cell levels, *daf-2* RNAi knockdown in the neurons of polyQ128 worms lowers the occurrence of total neuronal aberrations to levels observed in polyQ0 empty vector treated animals (Wald test, *p* < 0.01). However, *daf-2* RNAi polyQ128 touch response is still significantly lower than polyQ0 empty vector treated animals (Wald test, *p* < 0.01).

## Discussion

### Summary of findings

To begin to address the relationship between natural and proteotoxically challenged neuronal aging in a physiological context, we took advantage of high resolution *in vivo* analyses of *C. elegans* mechanosensory neuron morphology and function. We compared mid-life morphological and functional features of mechanosensory neurons that aged without, or with, the proteotoxic stressor polyQ128, under RNAi conditions that mimic systemic, including neurons, low or high activation of the insulin signaling pathway. Our data suggest that insulin signaling plays complex roles in neuronal maintenance in both healthy (polyQ0) and degenerate (polyQ128) neurons, with distinct outcomes on individual neurons notable even among the similar group of 6 mechanosensory neurons. Our data on individual neurons highlight remarkable neuronal diversity of responses to cellular signaling. Comparison of how the insulin signaling pathway impacts natural and proteotoxic-associated decline reveals some interesting differences in these two processes.

We conclude that RNAi down-regulation of the *daf-2* insulin receptor plays a role in morphological aging of mechanosensory neurons in both normally aging (polyQ0) and proteotoxically stressed (polyQ128) animals. Anterior and posterior neurons can be differentially affected by altered neuronal insulin signaling (Table 3). Importantly, our data suggest that decreased insulin signaling in normally aging systems (polyQ0) actually enhances some process aberrations in middle aged adults, with a focused impact on formation of ectopic branches on axons and dendrites. That *daf-2* mechanosensory function is improved raises the possibility that the structural aberrations (branching) we observe actually reflect consequences of a defensive response that enhances or protects neuronal health during aging (Figure 4). *age-1* may contribute partially to this process in polyQ0 animals, but evidence for the involvement of other pathway components is not compelling in our study. We also find that *daf-2* RNAi knockdown has distinctive impact on the proteotoxically stressed polyQ128 mechanosensory neurons: morphological aberrations are lower; and aggregates are fewer in number and smaller in size, with a striking subcellular restriction of aggregates to the cell body, as compared to empty vector controls. Somewhat surprisingly, other insulin signaling pathway disruptions have relatively modest impact on cell function, aggregate morphology, and aggregate distribution in polyQ128 animals; we found that *age-1* RNAi, which extends overall lifespan, did not confer dramatic changes in mechanosensory neurons, and often correlated with *daf-16* RNAi in inducing modest effects. Our data raise the possibility that, under conditions of extreme proteotoxic stress, neurons utilize a non-canonical *daf-2* pathway to enhance neuroprotection.

**Table 3:** **RNAi treatments targeting the insulin signaling pathway differentially effect polyQ128 specific mechanosensory neuronal aberrations**.

**Specific types of aberrations per**	**Cell type**	**RNAi treatment on polyQ128 animals**										
		**Empty vector (*N*_*anterior*_ = 226; *N*_*posterior*_ = 217)**	**(*daf-2 N*_*anterior*_ = 199; *N*_*posterior*_ = 202)**		***age-1* (*N*_*anterior*_ = 185; *N*_*posterior*_ = 185)**		***daf-18* (*N*_*anterior*_ = 92; *N*_*posterior*_ = 94)**		***akt-1* (*N*_*anterior*_ = 93; *N*_*posterior*_ = 92)**		***daf-16* (*N*_*anterior*_ = 143; *N*_*posterior*_ = 140)**	
		**Mean ± *SE***	**Mean ± *SE***	***p*-value**	**Mean ± *SE***	***p*-value**	**Mean ± *SE***	***p*-value**	**Mean ± *SE***	***p*-value**	**Mean ± *SE***	***p*-value**
Number of cell body outgrowths	Anterior	1.11 ± 0.05	0.86 ± 0.04	^*^0.00	1.32 ± 0.06	^*^0.01	1.21 ± 0.08	*n.s.*	1.24 ± 0.08	*n.s.*	1.29 ± 0.06	^*^0.03
	Posterior	0.01 ± 0.01	0.01 ± 0.03	*n.s.*	0.03 ± 0.03	*n.s.*	0.01 ± 0.01	*n.s.*	0.00 ± 0.00	*n.s.*	0.01 ± 0.01	*n.s.*
Number of branches	Anterior	0.15 ± 0.03	0.09 ± 0.02	^*^0.05	0.16 ± 0.03	*n.s.*	0.23 ± 0.05	*n.s.*	0.10 ± 0.03	*n.s.*	0.13 ± 0.03	*n.s.*
	Posterior	0.07 ± 0.02	0.05 ± 0.02	*n.s.*	0.08 ± 0.02	*n.s.*	0.05 ± 0.02	*n.s.*	0.03 ± 0.02	*n.s.*	0.05 ± 0.02	*n.s.*
Presence of punctae	Anterior	0.14 ± 0.02	0.04 ± 0.02	^*^0.00	0.37 ± 0.02	^*^0.00	0.27 ± 0.03	^*^0.02	0.39 ± 0.03	^*^0.00	0.33 ± 0.02	^*^0.00
	Posterior	0.71 ± 0.03	0.90 ± 0.03	^*^0.00	0.59 ± 0.04	^*^0.02	0.65 ± 0.05	*n.s.*	0.55 ± 0.05	^*^0.01	0.58 ± 0.04	^*^0.01
Presence of abnormal cell body	Anterior	0.13 ± 0.01	0.03 ± 0.01	^*^0.00	0.19 ± 0.01	*n.s.*	0.10 ± 0.02	*n.s.*	0.15 ± 0.02	*n.s.*	0.11 ± 0.01	*n.s.*
	Posterior	0.00 ± 0.01	0.02 ± 0.01	^*^0.02	0.03 ± 0.01	^*^0.01	0.02 ± 0.02	*n.s.*	0.00 ± 0.02	*n.s.*	0.03 ± 0.01	^*^0.02

*Table depicts mean number and significant difference for anterior (ALML or ALMR) and posterior (PLML or PLMR) mechanosensory neurons following RNAi treatment for each specific neuronal aberration observed (i.e., cell body outgrowths, process branching, presence of process punctae, and cell body abnormality). p-values indicate significance compared to appropriate empty vector control following generalized linear model analysis. Sample sizes for anterior (N_anterior_) and posterior (N_posterior_) neurons of each RNAi treatment are shown. Example outgrowths (A), branching (B), punctae (C), and abnormal cell body (D) are shown in Figure 4B*.

* *Denotes significance of *p* < 0.05*.

### Extending understanding of the influence of insulin signaling on healthy aging mechanosensory neurons

#### daf-2 insulin receptor

Mechanosensory neuron morphology changes with age in wildtype animals (Pan et al., 2011; Tank et al., 2011; Toth et al., 2012). Overall, genetic mutants and systemic RNAi treatments have suggested that *daf-2* mutants maintain youthful, aberration-free phenotypes longer than wildtype animals. Toth et al. (2012) distinguished among specific abnormality classes to measure decreases in cell body outgrowths and wavy process phenotypes, with a trend toward delaying branching in posterior mechanosensory neurons. Tank et al. (2011) showed that 10 day old *daf-2(e1370)* mutants had decreased process branching. Using a neuron-targeted *daf-2* RNAi approach, we measured an increase in neuronal aberrations at day 5 of adulthood, the vast majority of which are novel branches (Figures 4D,E). Because we find increased aberrations in *daf-2* RNAi coincident with a period of enhanced function (Figure 4A), we raise the question of whether mid-life branching might be a manifestation of a normal neuronal defense mechanism that actually improves sensory capacity, although this issue remains to be resolved with single animal functional imaging. This finding is also significant in that it suggests that all changes in morphology seen with age or treatments are not the same mechanistically and that some phenotypes, such as process branching, may be protective to neurons. Differences from other studies (Pan et al., 2011; Tank et al., 2011; Toth et al., 2012) could arise from differences in methods for reducing *daf-2* expression (with RNAi knockdown being distinct from modulation of specific amino acid resides in receptor reduction-of-function mutants) and different timing of scoring during adult life.

#### age-1 PI3 kinase and other insulin signaling molecules

We found that *age-1* RNAi modestly increased branching of mechanosensory neuron processes at day 5 of adult life, but did not alter touch sensitivity. The latter observation establishes that morphological phenotype and function are not always linked. *akt-1*, *daf-18*, and *daf-16* RNAi interventions did not alter the trajectory of age-associated morphological change in otherwise healthy neurons. The role of *age-1, akt-1*, and *daf-18* in morphological aging of the mechanosensory neurons has not been previously reported. Overall, *age-1* knockdown can have an effect on mechanosensory neuron morphological aging (Figures 4D,E) but impact on function is not large at mid-life. Other pathways that run in parallel may be important in mid-adult life (Tank et al., 2011).

#### daf-16

For natural aging of mechanosensory neurons, we find that *daf-16* RNAi exerts a small but statistically significant effect on day 5 adult posterior touch response, but not on aberrations. Tank et al. (2011) also came to the conclusion that aberrations for day 10 branching in a *daf-16* deletion mutant were similar to wildtype; whereas Pan et al. (2011) and Toth et al. (2012) noted a small increase in aberrations in *daf-16* mutants at days 9 and 10 (note the latter study, like ours, found no change at adult day 5). Still, time course studies do not support a profound impact of *daf-16* disruption on morphological aging of the mechanosensory neurons. *daf-16* appears to be needed for *daf-2(rf)*-mediated suppression of excess branching, though cell autonomy of this activity is disputed (Pan et al., 2011; Tank et al., 2011).

Overall, although compelling data support that insulin signaling a factor in natural aging of mechanosensory neurons, with reduced signaling correlating with reduced function, other pathways likely influence the process as well (Tank et al., 2011).

### Distinctive outcomes of daf-2 RNAi in the proteotoxically stressed huntington's disease model strain

#### daf-2

In middle-aged polyQ128 animals, we found that *daf-2* RNAi improved mechanosensory function, limited the number of morphological aberrations, and decreased overall aggregate number and size, compared to empty vector controls (Figure 5). Thus, reduced insulin receptor signaling through DAF-2 confers neuroprotection that is associated with diminished polyQ128 aggregate load in this model. Our data are consistent with studies in other disease models (Cohen et al., 2010) implicating *daf-2* in enhanced proteostasis during toxic protein challenge.

#### age-1 PI3 kinase and other insulin signaling molecules

We found that *age-1* RNAi modestly impairs touch sensitivity at a time point at which the mean number of aberrations and of aggregates are elevated relative to empty vector controls (Figure 4). Unexpectedly, *akt-1* and *daf-16* RNAi, which should have opposing impacts on the signal transduction pathway, induce similar outcomes in these proteotoxically challenged mechanosensory neurons. Because *age-1* RNAi [and sometimes *akt-1* (Hertweck et al., 2004)] extend lifespan (Table 2), dysfunction is not the anticipated outcome of such interventions. We emphasize two points here: first, our data raise the possibility that the most commonly outlined downstream pathway for DAF-2 signaling may not be the operative signaling pathway for the mechansensory neuron proteotoxicity pathway; and second, as it has previously been noted that other healthspan phenotypes differentially affected by *daf-2* vs. *age*-1 mutations. For example, in *age-1* mutants the biphasic profile for rate of increase in lipofuscin/age pigments during adulthood shows a temporal shift (delay in onset without change in time course), whereas for *daf-2* mutants the rate of lipofuscin accumulation remains low across adulthood (Gerstbrein et al., 2005). In other words, *daf-2* is more effective in preventing long term elevation in age pigment accumulation, while *age-1* delays onset of accumulation. This anomaly is a precedent for differential health outcomes following closely related insulin signaling pathway interventions.

In previous work, Morley et al. (2002) found that *C. elegans* expressing polyQ82 in body wall muscle show slower development of aggregates and motility defects with *age-1* RNAi and *age-1* mutants. While those authors showed the opposite effect of *age-1* RNAi on polyQ accumulation and toxicity compared to our study using a different cell type, they stressed the importance of threshold stresses in their interpretation. We note that our model carries an elevated polyglutamine load (polyQ128) as compared to polyQ82. In humans, longer polyglutamine expansion in huntingtin is well known to result in earlier onset of Huntington's disease and lower life expectancy.

#### daf-16

As expected, we observed *daf-16* RNAi confers diminished mechanosensory function together with increased anterior aberration and increased number and size of aggregates. Our results are consistent with previous studies showing that *daf-16* deficiency is associated with exacerbated polyQ128 proteotoxicity in young adult mechanosensory neurons (Parker et al., 2005, 2012; Lejeune et al., 2012) and with enhanced proteotoxicity in a polyQ82 model (Morley et al., 2002) and an Alzheimer's disease model (Cohen et al., 2009).

Overall, changes in anterior and posterior cell function, morphology, and protein aggregation load and localization (Figures 4, 5) correlate with whole animal observations (Figure 3) in both polyQ0 and polyQ128 strains. However, with polyQ128 *daf-16* RNAi we observed decreased anterior and posterior mechanosensory function (Figure 5A) and increased protein aggregate area and number in whole animal and both cell types (Figure 5D and Supplemental Figure 2), while only anterior mechanosensory neurons (ALML or ALMR) increased significantly in total neuronal aberrations (Figure 5B). This maintenance of neuronal morphology with worsened function and protein aggregation suggests other mechanisms can mediate these endpoints.

### *daf-2* RNAi uniquely changes distribution of polyQ128 aggregate load

A striking observation of *daf-2* RNAi animals is a profound difference in the distribution of polyQ128 aggregates (Figures 5E,F). *daf-2* RNAi is the only intervention we tested that induces localization of CFP-labeled polyQ128 aggregates nearly exclusively to the neuron cell body. Most aggregates we concentrated in only a few dots, which resembled perinuclear lysosomes. Although this subcellular domain restriction remains to be definitively identified, our observations suggest that for both anterior and posterior mechanosensory neurons, *daf-2* may exert neuroprotection by sequestering aggregates to prevent them from interfering with other cellular functions. The lower aggregate size suggests that enhanced degradation may occur when DAF-2 signaling is low. Our data also suggest that DAF-2 signaling could influence axonal transport of aggregate proteins or their retention in the cell body. Since we did not observe the cell body restricted pattern of *age-1* RNAi, a non-canonical downstream signaling pathway might be responsible for the observed effect.

### Neurons aging under extreme aggregate challenge exhibit differences from natural aging

It is striking that the *daf-2* RNAi polyQ128 neuronal aberration level is below that of polyQ128 empty vector controls (Figure 5B), and similar to that of polyQ0 empty vector controls (Figure 4C), while polyQ0 *daf-2* RNAi increases aberrations. Together with stresses induced by polyQ128, low insulin signaling (*daf-2* and *age-1* RNAi treatment) is associated both with protection from and an increase in morphological restructuring. Without extreme proteotoxic challenge at mid-life (as in polyQ0), however, neuronal aberrations are more apparent when insulin signaling is low. One possibility to explain these differences between *daf-2* RNAi in the naturally aging (polyQ0) and proteotoxically stressed (polyQ128) models is that hormetic consequences induced by polyQ128 are involved in suppression of aberrations. Alternatively, the aberrations in polyQ0 may be a manifestation of cellular maintenance that cannot be executed in the face of extreme polyQ128 challenge. An interesting question is whether additional neuron classes in the polyQ128 animals are affected by the expression of polyQ aggregates in the mechanosensory neurons or whether these effects are cell autonomous.

A second striking difference between natural aging and aging under extreme proteotoxic stress resides in *daf-16* RNAi treatment effects. In polyQ0 animals, *daf-16* RNAi impairs posterior touch sensation, but does not markedly alter neuronal morphology (only affecting anterior touch sensitivity). In contrast, in polyQ128 animals, *daf-16* RNAi results in both impaired touch response and increased neuronal aberrations. Also, *daf-16* RNAi in polyQ128, aggregate load increases and lifespan decreases proportionally more than in polyQ0. This suggests that polyQ128 expression in mechanosensory neurons induces stress signaling to other body tissues to disrupt the overall health of the animal.

A potential mechanism for the differences in effects of manipulating insulin signaling in polyQ0 and polyQ128 is increased basal insulin signaling in polyQ128 as a result of its protein load. However, we propose this is unlikely to be the mechanism underlying the observed differences. We see no difference in lifespan between empty vector control (L4440) treated polyQ0 and polyQ128 lifespan (Figure 2A). This is not surprising given that mechanosensory neurons do not seem to modulate lifespan (Chalfie et al., 1985). Further, microarray analysis of polyQ128 FACS-purified P_mec-3_ cells (mechanosensory neurons) showed no apparent dysregulation of genes in the insulin signaling pathway compared to non-toxic huntingtin:polyglutamine-expressing controls (Tourette and Neri, personal communication). Thus, the differences in neuron morphology and function seen in empty vector control treated polyQ0 and polyQ128 animals are likely due to some mechanism other than insulin signaling.

We have seen that with age, the effects on touch response and mechanosensory morphology are negatively correlated in polyQ0 and polyQ128 models (Vayndorf et al., in preparation). We showed that this correlation between increased morphological aberrations and decreased function remains in all RNAi treated polyQ128 animals (Figure 5). However, when polyQ0 animals receive RNAi treatment targeting components of the insulin signaling pathway, touch response and accumulation of aberrant morphology are no longer negatively correlated (Figure 4). Interestingly, while only *daf-2* RNAi showed overall significant effects on mechanosensory morphology and function in polyQ0 animals, RNAi treatment of polyQ128 animals yielded a higher number of significant changes. Also, we observed empty vector control polyQ128 animals decreased touch response and increased changes in neuronal morphology when compared to polyQ0 animals. Perhaps the decreased baseline neuronal function and worsened baseline neuronal morphology of the polyQ128 animals makes them more susceptible to changes in expression of insulin signaling pathway proteins, whereas naturally aging animals have more ability to compensate with other signaling pathways when there are changes in insulin signaling protein expression.

Overall, our findings suggest that improving neuronal aging outcomes and neuronal dysfunction associated with elevated protein aggregate stress may not be as simple as decreasing classical insulin signaling. This is not surprising because there are 40 insulin ligands with different expression levels in various cell types all vying for binding sites at the *C. elegans* insulin receptor, DAF-2. Others have shown that this insulin network is complex and works together to respond to varying stresses (Ritter et al., 2013), including protein misfolding and dysfunction like in our polyQ128 model. In addition, in mammalian and cell culture models, huntingtin is a phosphorylation substrate for AKT/PKB (Humbert et al., 2002; Dong et al., 2012). AKT can be modified and even cleaved into an inactive form in a rat model of Huntington's disease (Colin et al., 2005). Thus, in systems with endogenous huntingtin, unlike *C. elegans*, complex interactions between insulin signaling, expanded polyglutamine huntingtin aggregation, and neuronal morphology and function may be operative. Further genetic studies of the huntingtin polyQ128 model in *C. elegans* have the potential to elucidate mechanisms that influence morphological changes during neuronal aging.

### Conflict of interest statement

The authors declare that the research was conducted in the absence of any commercial or financial relationships that could be construed as a potential conflict of interest.
