# Supplementary material for: Insulin signaling in the aging of healthy and proteotoxically stressed mechanosensory neurons
Source: Front Genet. 2014 Jul 23;5:212. doi: 10.3389/fgene.2014.00212 (PMC4107846; doi:10.3389/fgene.2014.00212)
Supplement: Supplementary file 1 [file DataSheet1.PDF]

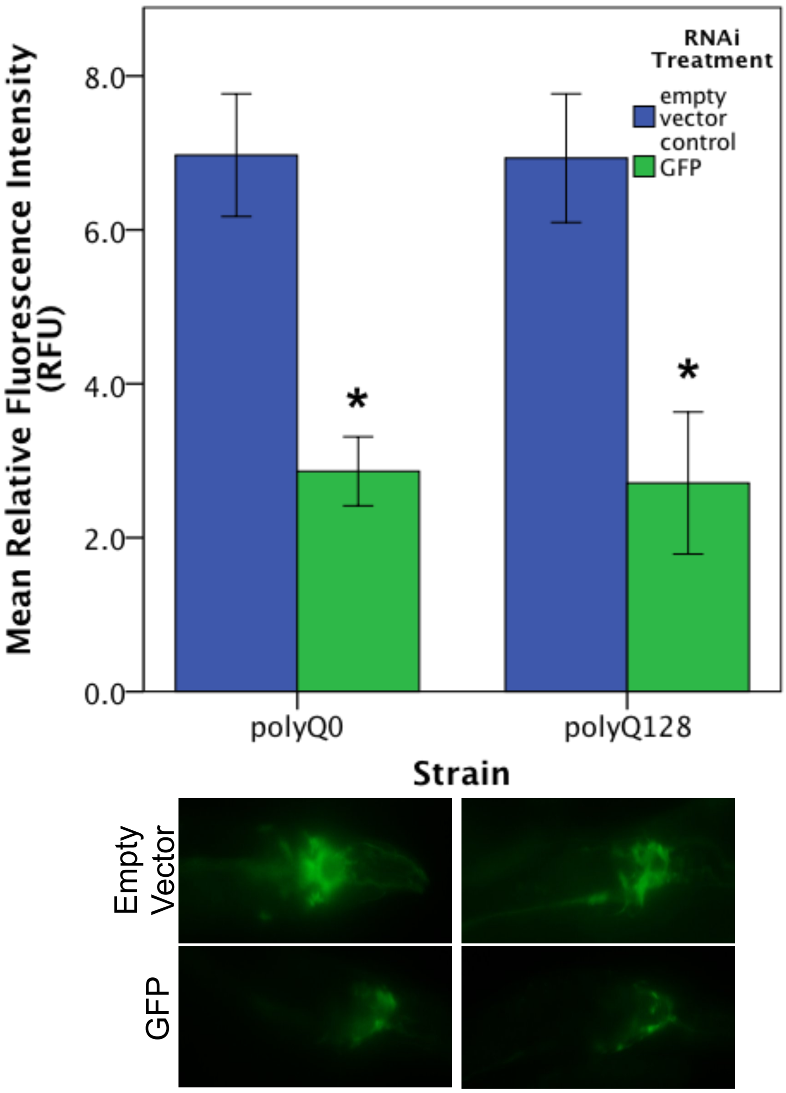

Supplemental Figure 1: polyQ0 and polyQ128 neurons are sensitive to RNAi treatments. Representative data and images of both polyQ0 and polyQ128 embryos treated with GFP RNAi treatment compared to empty vector controls. * denotes $p<0.001$ when compared to empty vector control following unpaired t-test. This experiment was repeated with similar results for each replicate of each RNAi experiment. Each bar represents mean relative fluorescence in arbitrary units (RFU) ± SE.

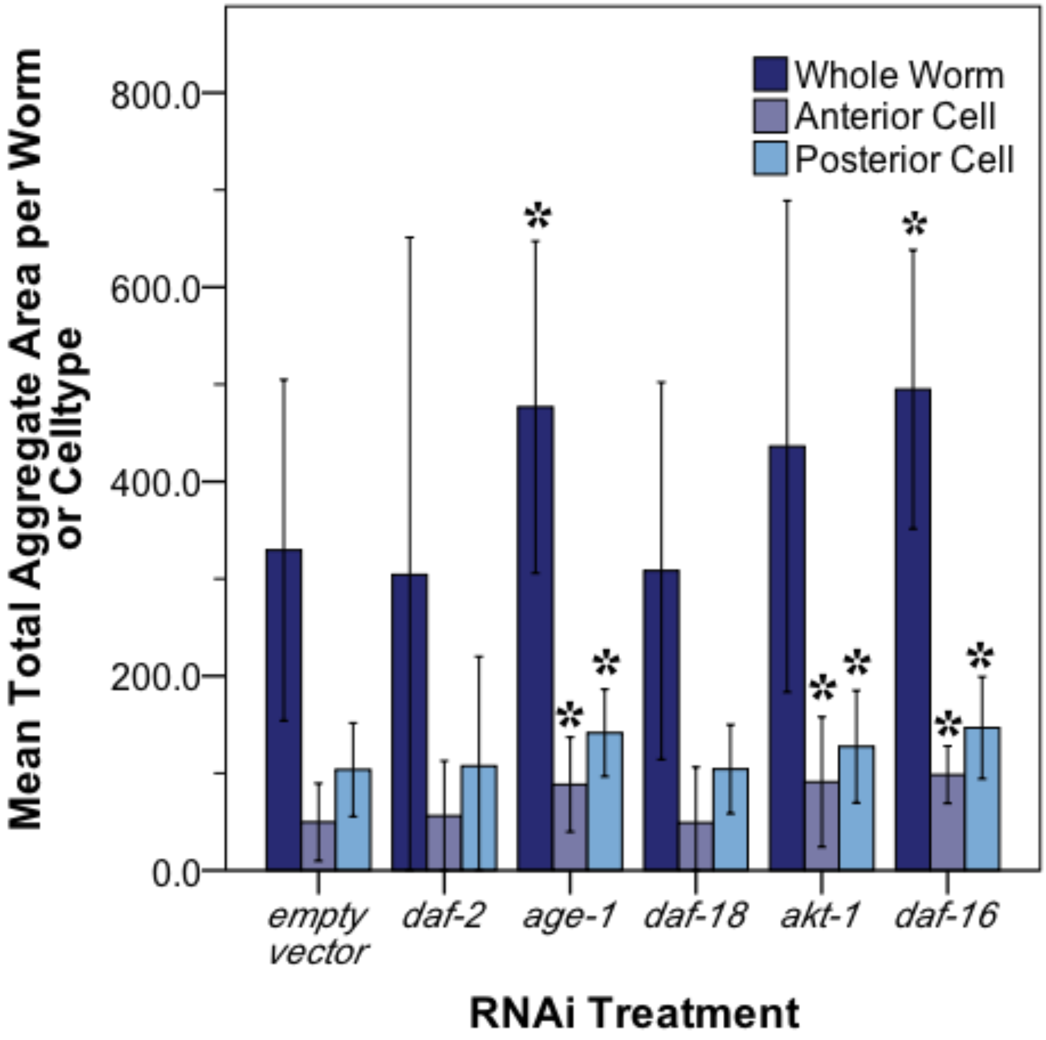

Supplemental Figure 2: Insulin signaling mediates poly128 aggregate area. Mean aggregate area per cell type and whole animal for each RNAi treatment. Aggregate area calculated using ImageJ. * denotes significance of <0.01 and # denotes significance of <0.10 relative to appropriate empty vector control following ANOVA and Tukey post-hoc analysis. Each bar represents mean ± SE.
